# Improved bioethanol production in an engineered *Kluyveromyces lactis* strain shifted from respiratory to fermentative metabolism by deletion of NDI1

**DOI:** 10.1111/1751-7915.12160

**Published:** 2014-09-03

**Authors:** María Isabel González-Siso, Alba Touriño, Ángel Vizoso, Ángel Pereira-Rodríguez, Esther Rodríguez-Belmonte, Manuel Becerra, María Esperanza Cerdán

**Affiliations:** Grupo de Investigación EXPRELA, Departamento de Bioloxía Celular e Molecular, Facultade de Ciencias, Universidade da CoruñaCampus de A Coruña, 15071-, A Coruña, Spain

## Abstract

In this paper, we report the metabolic engineering of the respiratory yeast *Kluyveromyces lactis* by construction and characterization of a null mutant (*Δklndi1)* in the single gene encoding a mitochondrial alternative internal dehydrogenase. Isolated mitochondria of the *Δklndi1* mutant show unaffected rate of oxidation of exogenous NADH, but no oxidation of matrix NADH; this confirms that *Kl*Ndi1p is the only internal NADH dehydrogenase in *K. lactis* mitochondria. Permeabilized cells of the *Δklndi1* mutant do not show oxidation of matrix NADH, which suggests that shuttle systems to transfer the NADH from mitochondrial matrix to cytosol, for being oxidized by external dehydrogenases, are not functional. The *Δklndi1* mutation decreases the chronological life span in absence of nutrients. The expression of *KlNDI1* is increased by glutathione reductase depletion. The *Δklndi1* mutation shifts the *K. lactis* metabolism from respiratory to fermentative: the *Δklndi1* strain shows reduced respiration rate and increased ethanol production from glucose, while it does not grow in non-fermentable carbon sources such as lactate. The biotechnological benefit of the *Δklndi1* mutant for bioethanol production from waste cheese whey lactose was proved.

## Introduction

In contrast to mammals, but similar to other eukaryotes like filamentous fungi or plants, in the inner mitochondrial membrane of yeasts, there are the so-called alternative dehydrogenases, or class-2 dehydrogenases, which are able to oxidize cytosolic and mitochondrial matrix NAD(P)H coenzymes and to transfer the electrons to ubiquinone. As opposed to complex I, these are non-proton-pumping, rotenone-insensitive and single polypeptide enzymes. Their precise physiological role in the different organisms has not been completely established, although it is known that they constitute a main entry point for electrons in the mitochondrial respiratory chain, being involved in the regulation of intracellular redox balance and energy production (Tarrío *et al*., 2006b). The characteristics of the mitochondrial alternative NAD(P)H dehydrogenases studied hitherto in yeasts are related to the preponderance of respiratory or fermentative metabolism in different species (González-Siso and Cerdán, 2007).

*Saccharomyces cerevisiae* and *Kluyveromyces lactis* are two yeasts that metabolize the glucose by oxidative and fermentative pathways, but *S. cerevisiae* is predominantly fermentative (Crabtree positive) and *K. lactis* is predominantly respiratory (Crabtree negative) (González-Siso *et al*., 2000). Both species lack complex I, which is present in strictly aerobic yeasts, and contain one internal alternative dehydrogenase and two external alternative dehydrogenases (Tarrío *et al*., 2006a; González-Siso and Cerdán, 2007).

The external alternative dehydrogenases show differential characteristics between the fermentative and the respiratory yeast. In *S. cerevisiae*, a decrease in expression of the external NADH dehydrogenases is observed at high glucose concentrations and this feature has been related to a higher amount of reoxidation of cytosolic NADH by the alcohol dehydrogenases and therefore with the prevalence of aerobic fermentation (the Crabtree-positive phenotype) (Luttik *et al*., 1998). In *K. lactis*, the ability of the external dehydrogenases to use NADPH, in addition to NADH, supports an increased use of the pentose phosphate pathway (PPP) to metabolize glucose in detriment of glycolysis (González-Siso *et al*., 1996a,b; Tarrío *et al*., 2005; 2006b).

Differently to mitochondria from plants and filamentous fungi, where complex I and internal alternative dehydrogenase coexist (MØller, 2002; Duarte and Videira, 2007), in *S. cerevisiae* and *K. lactis*, complex I is replaced by the alternative enzyme. Therefore, in these yeasts, the internal dehydrogenase constitutes the metabolic equivalent of complex I. In accordance with this function and in both yeasts, the expression of the alternative internal dehydrogenase is reduced at high glucose concentrations when fermentation is favoured (De Risi *et al*., 1997; Tarrío *et al*., 2005).

The ability to complement complex I defects has been proved for the *S. cerevisiae* alternative internal dehydrogenase, *Sc*Ndi1p (Yagi *et al*., 2006; Marella *et al*., 2008). Deficiency of complex I is one of the most frequent dysfunctions of the mitochondrial respiratory chain in human, often encompassing a large spectrum of clinical presentations, and is closely related to the aetiology of sporadic Parkinson's disease (Marella *et al*., 2008) and ageing (Balaban *et al*., 2005). The potential of the yeast *NDI1* gene in therapy of complex I defects has been demonstrated in rat animal models (Marella *et al*., 2008; 2010). This potential might be limited by the difference between the fermentative metabolism shown by *S. cerevisiae* cells and the oxidative metabolism shown by most tissues affected by complex I deficiency like nervous and cardiac; therefore, the use of the *NDI1* genes from Crabtree-negative yeasts could become advantageous (González-Siso *et al*., 2009).

In *S. cerevisiae*, *Sc*Ndi1p is also the closest homologue of mammalian AMID (apoptosis-inducing factor-homologous mitochondrion-associated inducer of death). Glucose repression of respiration (and also of antioxidant defences like the mitochondrial superoxide dismutase) has been proposed to be linked to the cell death process in yeast (Li *et al*., 2006). Besides, mitochondrial release (Cui *et al*., 2012) or overexpression of *Sc*Ndi1p (Li *et al*., 2006) has been directly related to apoptosis in *S. cerevisiae*.

In contraposition to *S. cerevisiae*, *K. lactis* shows an oxidative metabolism, is scarcely sensitive to glucose repression of respiration (González-Siso *et al*., 2000) and does not present an increase in longevity or a decrease in reactive oxygen species (ROS) formation when cultivated in lower glucose concentrations (Oliveira *et al*., 2008). These differences might involve the specific characteristics of the *NDI1* genes and their products of both yeasts, and their knowledge is important for their use in gene therapy of complex I disorders. Moreover, the regulation of *NDI1* genes is an important point to control the respiro-fermentative metabolism of yeasts and consequently their biotechnological use as cell factories (González-Siso *et al*., 2009) for recombinant protein production (González-Siso *et al*., 2008) or other productions, like bioethanol.

The above exposed reasons led us to study *Kl*Ndi1p, the alternative internal dehydrogenase of *K. lactis* mitochondria. In this work, we have constructed a null mutant in *KlNDI1*, the gene encoding this enzyme and used it to perform a functional analysis of *Kl*Ndi1p. The specific characteristics found in the null strain made it valuable to produce bioethanol from cheese whey lactose, a waste product, thus also proved in this work.

## Results and discussion

### Construction of the *Klndi1* null mutant

**Figure 1:**
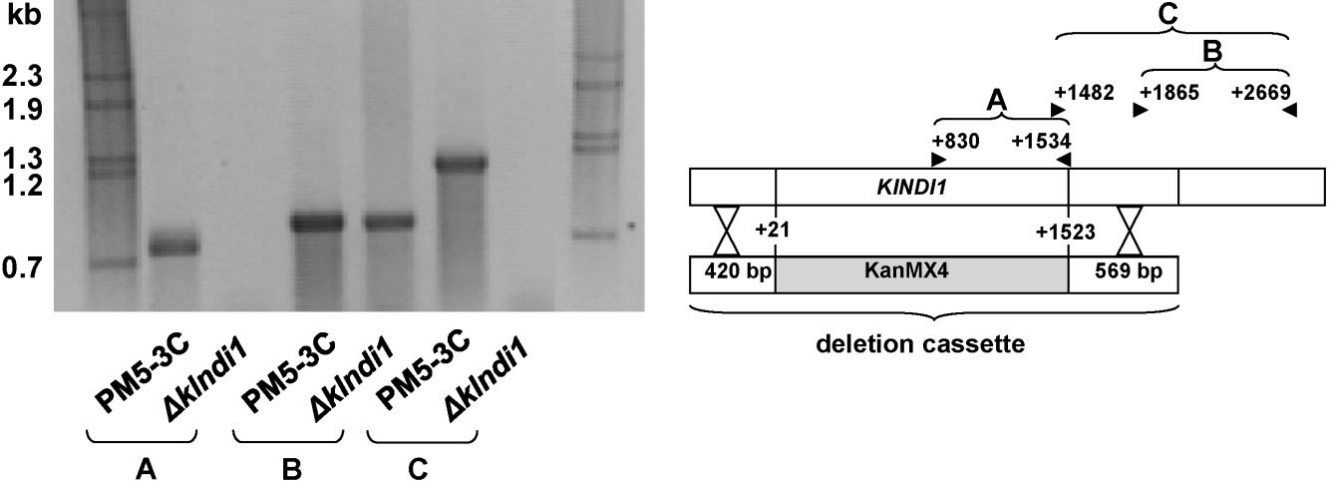
Analytical PCRs with genomic DNA of the wild type PM5-3c and *Δklndi1* mutant strains as template to prove the correctness of the deletion: (A) primers amplify from +830 to +1534 of *KlNDI1* sequence, (B) primers amplify from +1865 to +2669 of *KlNDI1* sequence and (C) primers amplify from +1482 to +2669 of *KlNDI1* sequence. Deleted region of the *KlNDI1* gene extends from +21 to +1523.

The *KlNDI1* gene was isolated from a *K. lactis* genomic DNA library as previously described (Tarrío *et al*., 2005) and subcloned in a commercial *S. cerevisiae* episomal vector. The resulting plasmid was used for the construction of the deletion cassette in which the coding region of *KlNDI1* was replaced by the KanMX4 module. The deletion cassette was introduced into the PM5-3C strain and the transformants selected for G418 (geneticin) resistance. Analytical polymerase chain reaction (PCR) confirmed the correct integration of the cassette in one of the two G418-resistant transformants obtained, which was named *Δklndi1* (Fig. 1) and used in the following analyses.

### Isolated mitochondria of the *Klndi1* null mutant show unaffected rate of oxidation of exogenous NADH but no oxidation of matrix NADH

Mitochondria from the *Δklndi1* mutant and from the corresponding wild-type PM5-3C strain were isolated and used to measure oxygen consumption as described in *Experimental procedures*. Both externally added NADH and a combination of malate and pyruvate were used as substrates. The combination of malate and pyruvate produces NADH in the mitochondrial matrix via the tricarboxylic acid cycle. Externally added NADH cannot pass through the membrane and enter the mitochondrial matrix; it can be oxidized by external alternative dehydrogenases in intact isolated yeast mitochondria.

**Figure 2:**
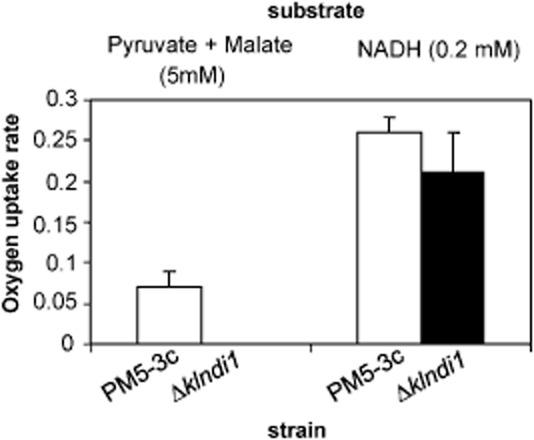
Exogenous NADH and pyruvate + malate oxidation by isolated mitochondria from *K. lactis* wild type (PM5-3c) and *Δklndi1* mutant. The oxygen uptake rates (μmol O_2_ min^−1^ mg protein^−1^) were measured in the presence of 0.25 mM ADP. Results are the mean ± standard deviation (SD) of two independent experiments.

Oxygen consumption by isolated mitochondria from the *Δklndi1* mutant compared with the wild-type strain (Fig. 2) shows that oxidation of matrix NADH is impaired in the mutant whereas oxidation of externally added NADH remains equal. This confirms that *Kl*Ndi1p, depleted in the mutant, is the single internal mitochondrial NADH : ubiquinone oxidoreductase in the mitochondria of *K. lactis*, and that activity of external alternative dehydrogenases is not affected by the *Δklndi1* mutation. A similar phenotype has been reported for isolated mitochondria from the *S. cerevisiae Δndi1* mutant (Marres *et al*., 2008; Bakker *et al*., 2000).

### In the *Klndi1* null mutant, there is no overexpression of the external alternative dehydrogenases

**Figure 3:**
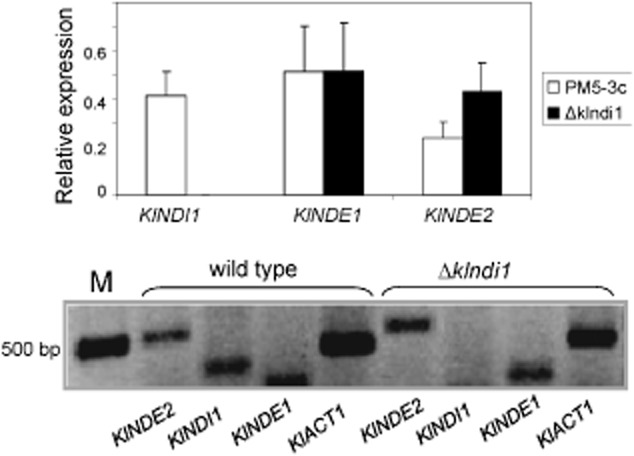
Expression (down) of the three *K. lactis* mitochondrial alternative dehydrogenase genes in the *Δklndi1* mutant compared with the wild-type strain (PM5-3c). Relative expression values (divided by *KlACT1*) are the mean ± standard deviation (SD) of four (wild type) or two (*Δklndi1* mutant) independent experiments (up).

Analysis of the expression of the three *K. lactis* mitochondrial alternative dehydrogenases in the *Δklndi1* mutant compared with the wild-type strain was done (Fig. 3). In the *Δklndi1* mutant, the band corresponding to the expression of the *KlNDI1* gene disappears as expected and quantification of the bands corresponding to *KlNDE1* and *KlNDE2* expression, responsible of cytosolic NADH oxidation, shows no statistically significant differences in reference to those in the wild-type strain (*P* values of 0.99 and 0.11, respectively, in the *t*-test performed to compare the means). This is in accordance with the above exposed results measuring oxygen consumption using NADH form cytoplasmic or matrix origin (Fig. 2) and corroborates that either expression or activity of external alternative dehydrogenases is not affected in the *Δklndi1* strain.

### The growth of the *Klndi1* null mutant in glucose is unaffected and in lactate is impaired

**Figure 4:**
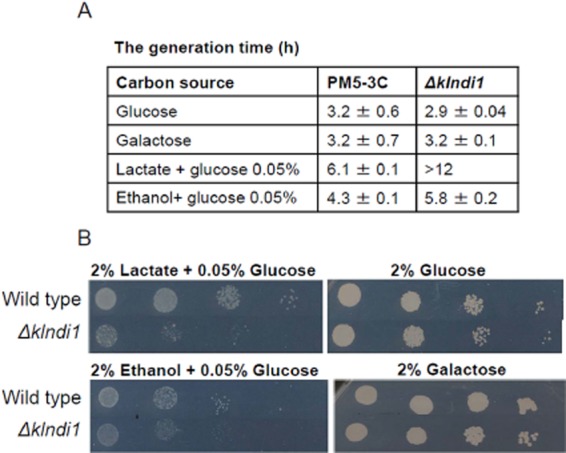
(A) The generation time (hours) of the *Δklndi1* mutant compared with the wild-type strain (PM5-3c) growing in liquid CM with the indicated carbon sources. Samples were taken each hour during the first 10 h of culture, inoculum at a OD_600_ of 0.1. These data are represented as mean ± standard deviation (SD) from two independent experiments. (B) Growth of the *Δklndi1* mutant compared with the PM5-3c strain (wild type for *NDI1*) in solid CM with the indicated carbon sources. Conditions as described in *Experimental procedures*. Photographs were taken after 1.5 days of growth on glucose, ethanol and lactate, and after 3 days of growth on galactose. *P < 0.05; **P < 0.01.

To test the effect of *KlNDI1* disruption on *K. lactis* metabolism, we examined the growth of the *Δklndi1* mutant in comparison with that of the parental PM5-3C strain in liquid (shake flask cultures) and solid complete medium (CM) containing various carbon sources under the conditions described in *Experimental procedures*. The mutant was no longer able to utilize non-fermentable substrates such as lactate while showed the same growth rate than the parental strain in fermentable substrates such us glucose (Fig. 4). A similar phenotype was reported for the *S. cerevisiae Δndi1* mutant (Marres *et al*., 2008; Bakker *et al*., 2001; Li *et al*., 2006). Also *Δndi1* mutants of both *K. lactis* (this work) and *S. cerevisiae* (Marres *et al*., 2008; Bakker *et al*., 2001) exhibited similar growth rate as wild-type cells in galactose. However, a difference is that ethanol sustained a more limited growth of the *Δklndi1* mutant than the wild type (Fig. 4), while growth of the *S. cerevisiae Δndi1* mutant in ethanol was unaffected with respect to the wild type (Marres *et al*., 2008; Bakker *et al*., 2001).

For growth on lactate and ethanol in the *Δklndi1* mutant, with the NAD^+^-linked steps of the Krebs cycle inhibited, the glyoxylate cycle and glucogenogenesis pathways have to be induced. The amount of ATP generated in this metabolic condition is lower than when the Krebs cycle and respiratory chain are fully operative as occurs in the wild-type strain, which may explain the reduced growth in non-fermentable carbon sources of the *Δklndi1* strain (Marres *et al*., 2008).

**Figure 5:**
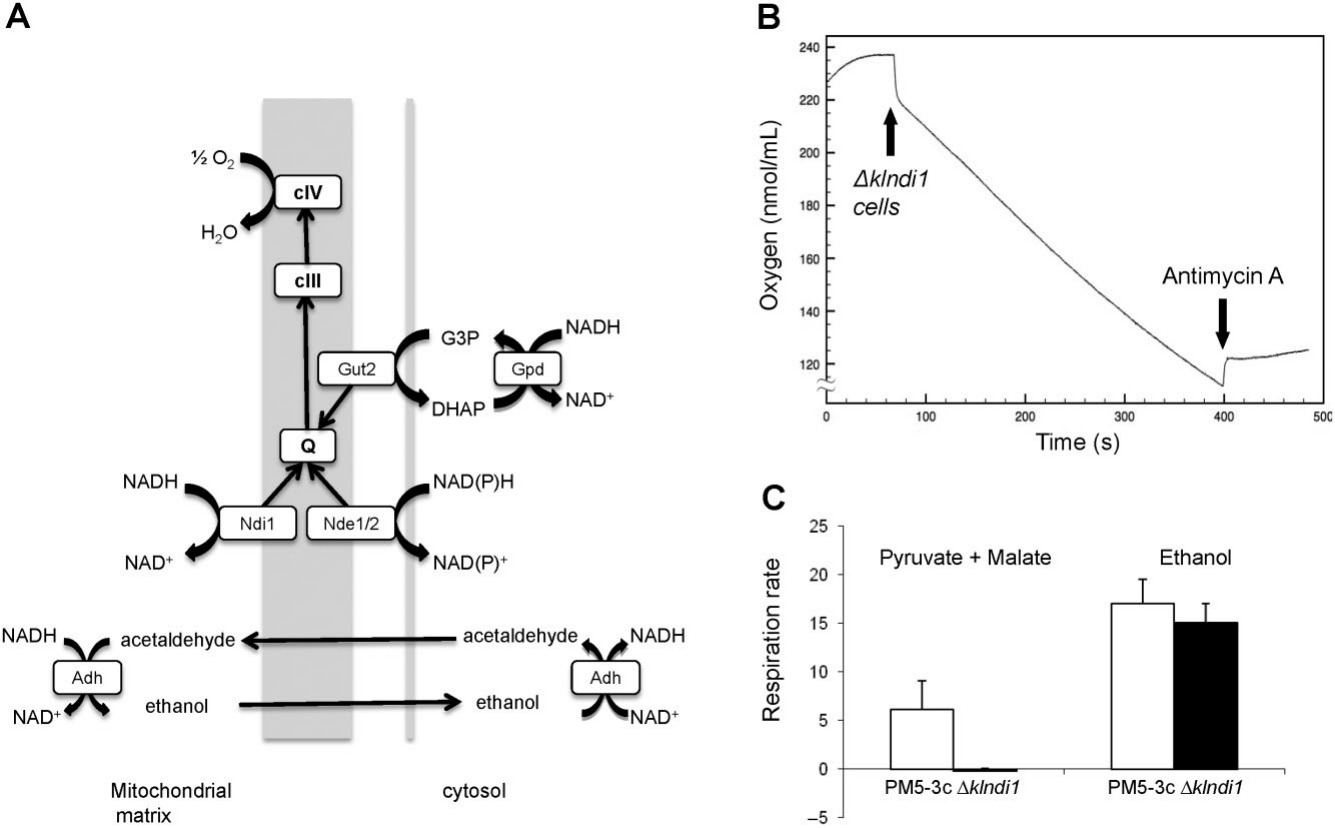
(A) Scheme of the *K. lactis* electron transport chain and cytosol-mitochondria NADH shuttle systems. Adh, alcohol dehydrogenases; Ndi1, internal NADH dehydrogenase; Nde1/2 external NAD(P)H dehydrogenases; Q, ubiquinone; DHAP, dihydroxy acetone phosphate; G3P, glycerol 3-phosphate; Gpd, glycerol 3-phosphate dehydrogenase; Gut2, FAD^+^-linked glycerol 3-phosphate dehydrogenase; cIII, complex III; cIV, complex IV. (B) Oxygen uptake by permeabilized cells of the *Δklndi1* mutant with 0.1 M ethanol as substrate stops upon antimycin A addition. (C) Respiration rate (nmoles O_2_ min^−1^ ml^−1^) by permeabilized cells of the *Δklndi1* mutant compared with the PM5-3c strain (wild type for *NDI1*) with the substrates pyruvate + malate and ethanol. Results are the mean ± standard deviation (SD) of three independent experiments. **P < 0.01.

The better growth of the *Δklndi1* strain observed in ethanol than lactate may also be explained by energetic and redox balances. For lactate utilization, three genes have been characterized so far in *K. lactis*, namely *KlJEN1*, *KlDLD* and *KlCYB2*, which encode a permease and two lactate ferricytochrome *c* oxidoreductases respectively (Rodicio and Heinisch, 2013). Lactate dissimilation by *S. cerevisiae* is also initiated by the oxidation of lactate to pyruvate via a cytochrome *c*-dependent mitochondrial lactate dehydrogenase and does not yield cytosolic NADH, while ethanol dissimilation by cytosolic alcohol dehydrogenases yields NADH that can be oxidized by the respiratory chain via both external mitochondrial dehydrogenases and the glycerol 3-phosphate shuttle, consisting of cytosolic NADH-linked glycerol 3-phosphate dehydrogenase and a membrane-bound FAD^+^-linked glycerol 3-phosphate : ubiquinone oxidoreductase (Bakker *et al*., 2001). Both cytosolic NADH-oxidation systems are in concerted regulation (Rigoulet *et al*., 2004). In *K. lactis*, two cytosolic isoforms of alcohol dehydrogenase, encoded by *KlADH1* and *KlADH2*, exist and these two mechanisms for cytosolic NADH oxidation and ATP generation in mitochondrial respiratory chain are also operative (González-Siso and Cerdán, 2007; Saliola *et al*., 2010), as schematized in Fig. 5A. These metabolic characteristics may explain the growth of the *Δklndi1* mutant in ethanol, limited but at a higher rate than in lactate.

### Permeabilized cells of the *Klndi1* null mutant show no oxidation of mitochondrial matrix NADH

In *S. cerevisiae*, there is experimental evidence that another important component of the redox-balancing system is the ethanol/acetaldehyde (Et–Ac) shuttle (Bakker *et al*., 2001). These metabolites, which can freely diffuse through mitochondrial membranes, are oxidized or reduced by cytosolic or mitochondrial alcohol dehydrogenases transferring the NADH excess from one cellular compartment to the other. The Et–Ac shuttle allows respiratory growth of the *S. cerevisiae Δndi1* mutant at the same rate than the wild-type strain in both glucose and ethanol as carbon sources. This is because matrix NADH is exchanged by the reversible interconversion of acetaldehyde into ethanol to the cytosol, where it can be oxidized by external mitochondrial dehydrogenases and the glycerol 3-phosphate shuttle. Nevertheless, this system is limited, since the mutant does not grow on acetate (Bakker *et al*., 2001). Saliola and colleagues (2010), based only on the fact that the addition of small amounts of ethanol, unable to sustain growth by itself, allowed a slight growth recovery in lactate medium of a *Δklndi1* mutant, have proposed that in *K. lactis*, the Et–Ac shuttle might be operative and could transfer the redox excess from the mitochondria to the cytoplasm, thus feeding the respiratory chain through the external dehydrogenases *Kl*Nde1p and *Kl*Nde2p (Fig. 5A).

To verify if the Et–Ac shuttle is active in *K. lactis* and, in complete absence of *KlNDI1* expression, might exchange matrix NADH to be oxidized by cytosolic systems, we measured oxygen consumption by permeabilized cells of *K. lactis* wild type and *Δklndi1* mutant with the combination of pyruvate and malate as substrate. The use of this system instead of isolated mitochondria allows to test reoxidation of internal produced NADH in conditions in which the cytoplasmic component of the shuttle is active. Blockage of oxygen consumption by antimycin A in *Δklndi1*-permeabilized cells proved that measured respiration rate corresponds to the mitochondrial respiratory chain (Fig. 5B). However, permeabilized cells of the *Δklndi1* mutant do not consume oxygen in the presence of the combination of pyruvate and malate as substrate (Fig. 5C). As a control, we measured oxygen consumption with ethanol as substrate and there is no significant difference between permeabilized cells of the *Δklndi1* mutant in comparison to the wild-type strain (Fig. 5C). This may be because ethanol metabolism produces NADH in the mitochondrial matrix but also in the cytosol since *K. lactis* has mitochondrial and cytosolic alcohol dehydrogenases and a functional glyoxylate cycle (Rodicio and Heinisch, 2013), and cytosolic NADH can be oxidized by the external alternative dehydrogenases of the respiratory chain. Therefore, the Et–Ac shuttle seems to be inactive in the *Δklndi1* mutant cells, at least in the conditions assayed.

### The expression of the *KlNDI1* gene is regulated by *KlGLR1* function

**Figure 6:**
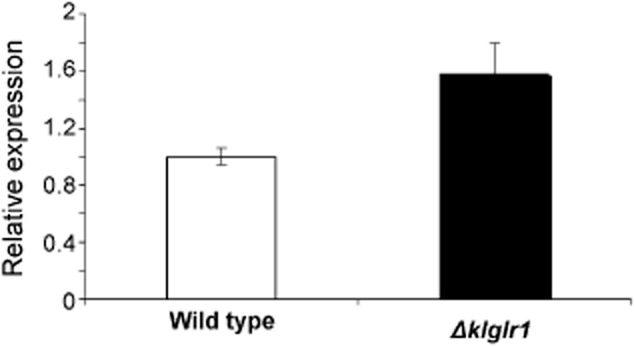
Relative expression of *KlNDI1* measured by qRT-PCR in the wild type NRRL-Y1140 and Δ*klglr1* mutant strains. Results are the mean ± standard deviation (SD) of two independent cultures analysed in duplicate. **P < 0.01.

In previous papers, we proved the involvement of *Kl*Glr1p, the *K. lactis* NADPH-dependent glutathione reductase (GLR), not only in the defence against oxidative stress but also in the regulation of the glucose metabolism in *K. lactis.* This second role was inferred both from the improved growth on glucose caused by the *Δklglr1* mutation and by the increased GLR activity in the *Δklgcr1* strain (García-Leiro *et al*., 2010a) being *Kl*Gcr1p a positive transcriptional regulator of glycolytic genes, as well as by the fact that enzymatic activities of GLR and glucose 6-phosphate dehydrogenase (G6PDH) from the PPP are positively correlated (Tarrío *et al*., 2008). Thus, G6PDH is positively regulated by an active respiratory chain and GLR plays a role in the reoxidation of the NADPH from the PPP in these conditions (Tarrío *et al*., 2008). A further proteomic analysis provided additional results; H_2_O_2_ addition causes downregulation of enzymes from the glycolytic pathway and Krebs cycle in wild-type *K. lactis*, whereas *Δklglr1* deletion prevents this effect and actually causes upregulation of the glycolytic, Krebs cycle and oxidative PPPs, which led us to propose that the redox imbalance produced by the *Δklglr1* deletion controls glucose metabolic fluxes in *K. lactis* (García-Leiro *et al*., 2010b). New data obtained in this work also support this proposal (Fig. 6). Thus, RNA was extracted from cells grown in CM with 2% glucose (OD_600_ = 0.8) and *KlNDI1* mRNA levels were measured by quantitative reverse transcription polymerase chain reaction (qRT-PCR) in a *Δklglr1* mutant (García-Leiro *et al*., 2010a) and the isogenic parental strain. Significant differences were found: *KlNDI1* mRNA levels being 1.58-fold higher in the Δ*klglr1* mutant. The interpretation of these results is that GLR depletion, by reducing NADPH oxidation rate and therefore NADP^+^ availability, necessary for G6PDH activity, could redirect glucose flow from the PPP which is preponderant in *K. lactis* for glucose metabolism (González-Siso *et al*., 2000; 2009) to glycolysis and Krebs cycle, which will be associated to increased *KlNDI1* expression.

### The chronological life span of the *Klndi1* null mutant is decreased

It has been reported that *Sc*Ndi1p is the closest homologue of mammalian AMID in yeast and is involved in chronological ageing (Li *et al*., 2006). Overexpression of *Sc*Ndi1p causes apoptosis-like cell death. This effect can be repressed by increased respiration on glucose-limited media, being the apoptotic effect of *ScNDI1* overexpression associated with increased production of ROS in mitochondria (Li *et al*., 2006). We have determined that also in *K. lactis*, the closest homologue to human AMID is *Kl*Ndi1p (González-Siso and Cerdán, 2007), and therefore studied its putative role in ageing as following described.

**Figure 7:**
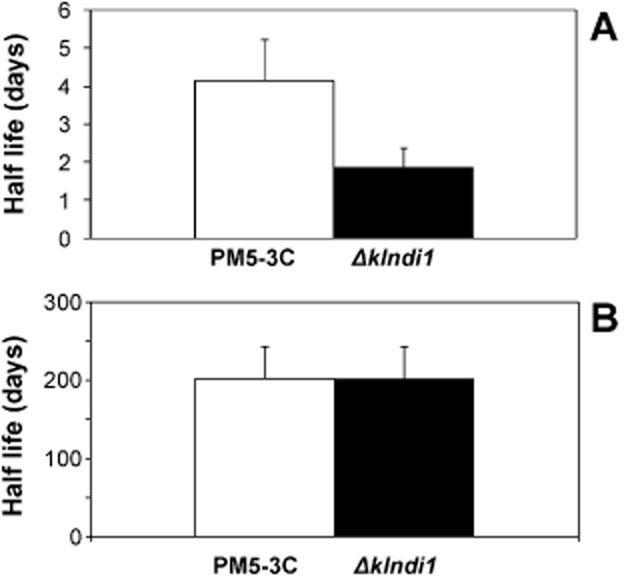
Life span of the wild type PM5-3c and *Δklndi1* mutant strains. Conditions as described in *Experimental procedures*. (A) Survival in distilled water. Results are the average of four independent cultures. (B) Survival with culture medium renewal. Results are the average of two independent cultures. **P < 0.01.

Chronological life span of the *Δklndi1* mutant and parental strains was measured using the protocols described in the *Experimental procedures*. For cells grown in a CM and further maintained in distilled water, significant differences in viability (quantified as half-life) were found between the *Δklndi1* mutant and wild-type strain (Fig. 7A), the viability being decreased in the mutant. This situation is different to the one reported in *S. cerevisiae* where disruption of *NDI1* decreases ROS production and elongates the chronological life span of yeast, accompanied by the loss of survival fitness (Li *et al*., 2006). However, considering that these authors measured life span with culture medium renewal each 3 days, while our conditions are survival in absence of nutrients (distilled water), we also measured the life span of the *Δklndi1* mutant and parental strains using the same protocol than Li and colleagues (2006) without finding increased life span in the mutant (Fig. 7B). In the latter conditions but before medium renewal, there was neither significant difference in ROS levels (in exponential and stationary culture phase, measured as in Tarrío *et al*., 2008), nor DNA and nuclear fragmentation between the *Δklndi1* mutant and wild-type strain (data not shown). The differences caused by *NDI1* deletion in the life span of the two species might be related to differences in respiratory metabolism and ROS production (reviewed in González-Siso *et al*., 2009), and their dependence on *NDI1* function, but further work is necessary to clarify this issue.

Interestingly, the *Δnde1* mutation has been reported to shorten replicative life span in *S. cerevisiae* and the authors proposed that this effect results from multiple mechanisms including the low respiration rate and low energy production rather than just from a ROS-dependent pathway; however, regarding chronological life span, measured in rich medium, Δ*nde1* cells were able to survive better than wild-type cells in the stationary phase (Hacioglu *et al*., 2012).

### The *Klndi1* null mutant shows overall reduced respiration rate and increased ethanol production from glucose

**Figure 8:**
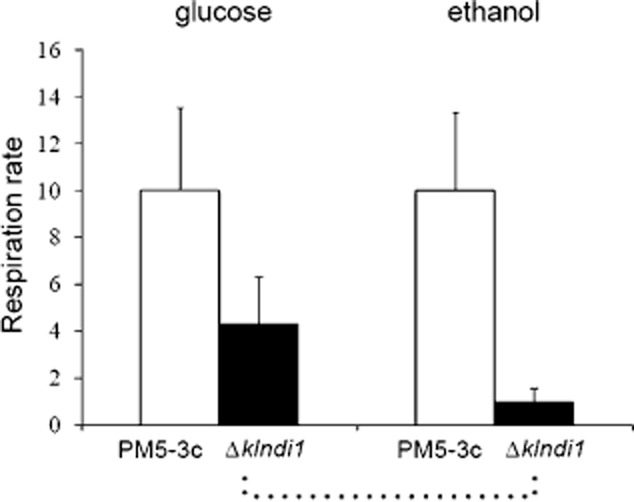
Cell respiration rate (nmol O_2_ ml^−1^ min^−1^ OD_600_^−1^) of *K. lactis* PM5-3c and *Δklndi1* mutant cells cultured in glucose media and with glucose or ethanol as substrates. Results are the mean ± standard deviation (SD) of four independent cultures analysed in duplicate. *P < 0.05; **P < 0.01.

To increase supporting evidence of the effect of *KlNDI1* disruption on yeast metabolism, we measured the overall respiration rate, with glucose and ethanol as substrates, in cells of the *Δklndi1* mutant and parental strains, sampled at two OD_600_ values (2 and 6) from glucose (YPD) cultures. Figure 8 shows that overall respiration rate of the mutant compared with the parental strain is reduced both with glucose or ethanol as substrates. There is also a significant difference in cell respiration rate of the *Δklndi1* mutant (but not in the case of the wild-type strain) being lower in ethanol compared with glucose as substrate.

We also measured ethanol production in glucose liquid cultures of the *Δklndi1* mutant and wild-type strains, obtaining increased ethanol production in the mutant. Thus, after 2 days of culture in CM with 2% glucose, when glucose concentration in the medium was lower than 1 g l^−1^ and OD_600_ about 10, ethanol in the culture medium was higher for the mutant strain than for the PM5-3C strain (1 ± 0.18 g l^−1^ versus 3.3 ± 0.17 g l^−1^, *n* = 2). Similar results were obtained in a *Δklndi1* mutant obtained in a different genetic background (Saliola *et al*., 2010). Higher ethanol levels are due to the lower respiration rate of the mutant (Fig. 8) and, moreover, to ethanol accumulation due to its limited use as carbon source in the *Δklndi1* mutant, also proved in this work (Fig. 4).

Both respiration rate and ethanol production measurements indicate a more fermentative glucose metabolism in the case of the *Δklndi1* mutant compared with the parental strain that has biotechnological application in whey utilization as following reported.

### Ethanol production from cheese whey lactose by the *Klndi1* null mutant

Actual market trends point to a worldwide gradual increase in cheese production that generates whey as waste product (9 kg of whey per 1 kg of cheese). Whey disposal constitutes a pollution problem even though systems for its treatment have been developed. The most abundant component of cheese whey is the disaccharide lactose that remains in the permeate after whey ultrafiltration for protein recovery. The treatment of whey permeate by fermenting lactose to ethanol has received wide attention to date and there are industrial plants in operation (Guimarães *et al*., 2010). However, the development of a high productivity lactose fermenting process continues to be of prime importance. *Saccharomyces cerevisiae* is unable to ferment directly lactose to ethanol and the microorganisms mainly used for this purpose are selected *Kluyveromyces* spp. To reduce distillation costs, it is recommended to use concentrated whey up to 120 g lactose l^−1^ (González-Siso, 1996; Arif *et al*., 2008).

**Figure 9:**
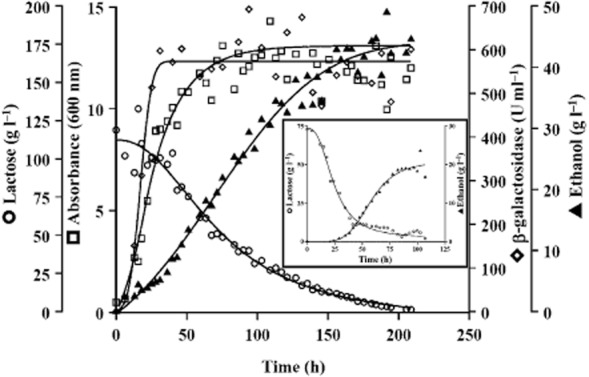
Parameters of the *Δklndi1* respiratory mutant growing in a 2 l bioreactor with threefold concentrated cheese whey permeate as substrate. Insert figure depicts the parameters of NRRL-Y1140 growing in a 2 l bioreactor with twofold concentrated cheese whey permeate as substrate. Conditions as described in *Experimental procedures*.

In this work, we took advantage of the predominantly fermentative metabolism shown by the *Δklndi1* mutant and assayed the use of this strain for the bioconversion of lactose to ethanol in concentrated cheese whey permeate. Figure 9 shows that the *Δklndi1* mutant, grown in the fermenter culture conditions described in *Experimental procedures*, fully degrades the lactose in 8–9 days and produces up to 50 g l^−1^ ethanol which gives a yield (80.1% of the theoretical maximum of 4 mol of ethanol from 1 mol of lactose) close to the maximum reported for industrial strains (Della-Bianca *et al*., 2013; Diniz *et al*., 2007), and higher than the NRRL-Y1140 laboratory strain that is wild type for the *KlNDI1* gene (the yield ranged between 62.6% and 50% of the theoretical maximum in the same conditions, according to the highest value and the value adjusted to the curve respectively). Although the yield obtained with the NRRL-Y1140 strain seems high for a respiratory yeast, the distribution between respiratory and fermentative metabolism in wild-type *K. lactis* is dependent on oxygen availability (González-Siso *et al*., 1996a; Guimarães *et al*., 2010) and low levels of oxygen in these cultures favour fermentation. Similar ethanol yields in whey than those reached by the NRRL-Y1140 strain in this work were obtained by other authors in fermentative conditions with other *Kluyveromyces* strains (Szczodrak *et al*., 1997; Guimarães *et al*., 2010).

Once we proved the ethanol production performance of the Δ*klndi1* mutant, we decided to transform this strain with the multicopy plasmid pSPGK1-LAC4 (Becerra *et al*., 2001) to improve its capability to grow in lactose and therefore ethanol production. The pSPGK1-LAC4 plasmid expresses the *LAC4* gene that codes for *K. lactis* beta-galactosidase. The results show that the Δ*klndi1* mutant, in the sealed tube conditions described in *Experimental procedures*, degrades the 79% of the lactose in 4 days (a 56% more than the untransformed strain) producing ethanol with a yield (quantified as percentage of the theoretical maximum of 4 mol of ethanol from 1 mol of lactose) 16.4% higher for the transformed versus untransformed strain. Overexpression of beta-galactosidase increases both lactose consumption rate and yield of ethanol from lactose.

These results support the biotechnological use of *Δklndi1* and its derivative pSPGK1-LAC4 transformants for efficient fermentation of cheese whey to bioethanol.

## Conclusions

In this paper, we have constructed and characterized an engineered *K. lactis* strain by deletion of the *KlNDI1* gene. We proved that *KlNDI1* encodes the single internal NADH : ubiquinone oxidoreductase in the mitochondria of *K. lactis* (*Kl*Ndi1p), and that activity of external alternative dehydrogenases is not affected by the *Δklndi1* mutation. Similarly to data previously reported for the null in *S. cerevisiae*, the *Δklndi1* mutant grows at the same rate than the wild-type strain in glucose but is unable to grow in lactate. Differently to the phenotypes reported for the *Δndi1* in *S. cerevisiae*, the *Δklndi1* strain grows in ethanol with a lower rate and shows reduced chronological life span in absence of nutrients compared with the wild-type strain. Besides, shuttle systems transferring NADH from the mitochondrial matrix to the cytosol for oxidation by external dehydrogenases do not operate in permeabilized *K. lactis* cells. As a result of multiple functional differences, the *Δklndi1* mutation shifts glucose metabolism to fermentative, thus increasing the performance of the *Δklndi1* strain for bioethanol production from lactose present in cheese whey. This is a biotechnological advantage versus *S. cerevisiae*, which is unable to metabolize lactose, and versus *K. lactis* expressing *NDI1*, which is highly respiratory. The *Δklndi1* strain combines three important characteristics: the ability to use lactose, high levels of ethanol production and low use of the ethanol produced as carbon source.

## Experimental procedures

### Strains, media and culture conditions

The *K. lactis* strain PM5-3C (*MATa uraA Rag*^+^) (Wésolowski-Louvel *et al*., 1992) was used.

Growth and handling of yeasts were carried out according to standard procedures (Kaiser *et al*., 1994). The yeast cells were cultivated, unless otherwise stated, in Erlenmeyer flasks at 30°C and 150–250 r.p.m. in the synthetic CM (Zitomer and Hall, 1976), the dropout medium CM-ura (without uracil) or YP medium (2% bactopeptone, 1% yeast extract) containing one of the following carbon sources: 0.1–2% glucose, 2% lactate, 2% ethanol and 2% galactose. Lactate and ethanol media were supplemented with 0.05% glucose. The flasks were filled with 40% volume of culture medium. Solid growth media also contained 1.5% agar.

For growth analysis on solid CM, cells from overnight Erlenmeyer cultures in the same medium were diluted to OD_600_ = 0.1 and then several serial 1:10 dilutions were made. Drops (0.005 ml) of the dilutions were added to the solid media, incubated at 30°C and photographed after 1.5 and 3 days.

### Construction of null mutant

Deletion of nucleotides +21 to +1523 in the *KlNDI1* gene (Tarrío *et al*., 2005) was carried out by the one-step method (Rothstein, 1991). The template of the deletion cassette was constructed as described in Zaragoza (2003) in the plasmid YEplac195, and included the kanamycin resistance gene (the *kanMX4* module) flanked by 420 bp homologous to the *KlNDI1* 5′ region and 569 bp homologous to the *KlNDI1* 3′ region. The deletion cassette was PCR amplified and the product transformed to the *K. lactis* wild-type strain PM5-3C. For selection of transformants, geneticin was added at a final concentration of 0.3 mg ml^−1^ to YPD medium. Correct *KlNDI1* gene deletion was verified by analytical PCR. Genomic DNA as template and several combinations of inward primers, binding outside the deletion cassette, and outward primers, binding within the deleted region, were used. Also, the absence of *KlNDI1* mRNA in the null mutant was verified by reverse transcriptase-polymerase chain reaction (RT-PCR).

### Isolation of RNA, production of cDNA and RT-PCR

The kits NucleoSpin (Macherey-Nagel) and SuperScript II (Promega) were used for RNA extraction from the *K. lactis* cells and for cDNA synthesis and RT-PCR, respectively, following the instructions of the suppliers. Twenty cycles of PCR were performed with primers, which amplified the cDNA from positions +1020 to +1380 of the *KlNDI1* cds, +1088 to +1603 of the *KlNDE2* cds and +31 to +315 of the *KlNDE1* cds. *KlNDE1 a*nd *KlNDE2* are the genes encoding the two *K. lactis* external alternative dehydrogenases (Tarrío *et al*., 2005; 2006b). The gene of the *K. lactis* actin (KLLA0D05357g) was used as control (positions +1290 to +1728 of the cds). Quantification of the products, separated on agarose gels, stained with ethidium bromide and photographed with a digital system, was performed in tagged image file format with densitometry analysis software (ImageMaster Total Laboratory, version 2.00, GE Healthcare).

### Analysis of expression by qRT-PCR

Total RNA isolated using the AURUM kit (Bio-Rad) was converted into cDNA and labelled with the KAPA SYBR FAST universal one-step qRT-PCR kit (Kappa Biosystems, Woburn, MA, USA).

PCR primers for *KlNDI1* were designed with the tool ‘Primer Blast’ at NCBI to generate 80–120 base pairs amplicon. Primer length of 18–24 bp, Tm of 59 or 60°C and G/C content not higher than 50% were selected. The sequence of primers chosen is GAATACTTGGCTAAGGTCTTCGAC (forward) and CTTGAATGGCTTGAAACCGTTTTC (reverse). The specificity of the primers for the *KlNDI1* cds was verified.

The ECO Real-Time PCR System was used for the experiments (Illumina, San Diego, CA, USA) and calculations were made by the 2-ΔΔCt method (Livak and Schmittgen, 2001). Two independent cultures and RNA extractions were assayed for each strain or condition. The mRNA levels of the *KlNDI1* gene were corrected by the geometric mean of the mRNA levels of the gene *ACT1*, a control gene also used for RT-PCR experiments as above described, which was previously verified to be constitutive in the assayed conditions. A *t*-test was applied to evaluate the differences between ΔCt values (Ct values normalized with reference genes) of control and treated samples with a *P* value of 0.05.

### Cell respiration rate

The respiration rate of *K. lactis* PM5-3c and *Klndi1* null mutant cells was measured at 25°C using a Clark-type oxygen electrode (Hansatech) following the procedure previously described (García-Leiro *et al*., 2010a). The cells grown in liquid YPD medium were collected by centrifugation (previously OD_600_ was measured) and suspended in one-tenth volume of 50 mM potassium phosphate (pH 6.5) buffer with 0.1 M glucose or ethanol. 50–100 μl of the cell suspension was introduced into the chamber filled with 1 ml of the same buffer and the amount of oxygen consumed was recorded. Data are expressed as nmol O_2_ ml^−1^ min^−1^ OD_600_^−1^.

### Mitochondrial respiration rate in permeabilized cells

Five millilitres of *K. lactis* cells of the wild-type PM5-3c and *Klndi1* null mutant strains grown in liquid medium (10 ml of culture in a 50 ml Erlenmeyer flask incubated at 28°C and 250 r.p.m.) up to OD_600_ = 1 were collected by centrifugation and suspended in 5 ml of phosphate buffer 25 mM pH = 7.2 with MgCl_2_ 5 mM and sorbitol 0.65 M. For permeabilization 0.1 ml of 50× protease inhibitor Complete Mini, EDTA Free (Roche) and 0.025 ml of digitonin 10 mM were added, tubes were gentle agitated during 1 min, cells collected again by centrifugation and suspended in 0.5 ml of the same buffer.

The respiration rate (oxygen uptake) of permeabilized cells was measured at 28°C using a Clark-type oxygen electrode (Hansatech). In the electrode cuvette, 0.8 ml of buffer was added plus 0.05 ml of adenosine diphosphate (ADP) 50 mM and 0.05 ml of the substrate (1 M pyruvate-malate or 2 M ethanol). After baseline stabilization, 0.1 ml of the permeabilized cell suspension was added and the amount of oxygen consumed was recorded. The basal respiration of cells in the same conditions but without substrate was subtracted. Data are expressed as nmol O_2_ ml^−1^ min^−1^. All respiration was found to be sensitive to antimycin A.

### Glucose, lactose and ethanol

Analyses were done as previously described (González-Siso and Suárez Doval, 1994) with a refractive index detector coupled to an isocratic high-performance liquid chromatography system operating with a Sugar-Pack column (Waters, Millipore) at 90°C, Milli-Q water was the mobile phase at 0.5 ml min^–1^.

### DNA and nuclear fragmentation

To evaluate genomic DNA and nuclear integrity, cultures of *K. lactis* PM5-3c and *Klndi1* null mutant in liquid YPD media (2% glucose) were inoculated at OD_600_ = 0.1 from fresh overnight pre-cultures and every day until 4 days an aliquot was taken. Genomic DNA was extracted and visualized after electrophoresis on agarose gels and ethidium bromide staining. Nuclear staining was performed, after fixing the yeast cells with 2.5% glutaraldehyde on ice for 10 min, with 10 μg ml^−1^ propidium iodide and 2.5 μg ml^−1^ 4,6-diamidino-2-phenylindole. The stained cells were viewed with a fluorescent microscope and photographed with a digital camera.

### Life span

Chronological life span of *K. lactis* PM5-3c and *Δklndi1* mutant was determined by measurement of colony-forming ability [colony-forming units (cfu)]. Cultures in liquid YPD and CM media with 2% glucose (two cultures in YPD and two cultures in CM) were inoculated at OD_600_ = 0.1 from fresh overnight pre-cultures in the same medium; after 2 days of growth, the cells were washed and transferred to distilled water at OD_600_ = 1 and further incubated in the same conditions (at 30°C and 150–250 r.p.m.). The first sample was taken at the moment of transfer and considered 100% viability. Aliquots of the cultures containing approximately 100 cells were plated to YPD medium. Every 2 days, new samples were taken to score viability until less than 10% of the culture was viable. The half-life of each strain was calculated by exponential fit of the data with Excel software.

Survival of *K. lactis* PM5-3c and *Δklndi1* mutant was also determined with culture medium renewal (Li *et al*., 2006). Percentage of viable versus total cell number was measured, by methylene blue staining and counting in Neubauer chamber. Only unviable cells are stained by this dye. Cultures were performed as described above in YPD but cells were transferred to fresh medium every 3–4 days during 6 weeks. Just before medium renewal, samples were taken and cellular viability was measured.

### Isolation of mitochondria

*Kluyveromyces lactis* mitochondria were isolated as previously described (Tarrío *et al*., 2005; 2006b). Cells were aerobically grown in CM with 1% glucose as carbon source.

### Oxygen uptake studies with mitochondrial preparations

Substrate-dependent oxygen consumption rates of freshly isolated mitochondria were determined at 30°C using a Clark-type oxygen electrode (Hansatech) as previously described (Tarrío *et al*., 2005; 2006b). Respiratory substrates were NADH (0.2 mM) and pyruvate + L-malate (5 mM), which generate intramitochondrial NADH. Measurements were made in the absence or presence of 0.25 mM ADP, and all respiration was found to be sensitive to antimycin A.

### Bioethanol production from cheese whey

Cheese whey ultrafiltrated permeate, threefold concentrated, was obtained from a local cheese factory (Queizúar S.L.). Cultures of wild type and *Klndi1* null mutant strains were performed in a 2 l vessel bioreactor (Biostat MD). Preinocula were grown in YPD at 30°C. Temperature, pH, agitation and aeration were maintained at 30°C, 5.6, 500 r.p.m. and 1–1.3 l min^−1^ respectively for 40 h when aeration was stopped and agitation reduced to 150 r.p.m. Samples were taken at determined time intervals for analysis of growth (OD_600_), beta-galactosidase activity, lactose and ethanol.

For cultures in sealed tubes, preinocula were grown in 500 ml flasks at 30°C in 200 ml YPD. When cells reached an OD_600_ = 3 at 600 nm, they were centrifuged and added as inoculum to 100 ml sealed tubes containing 40 ml of three-times concentrated cheese whey permeate. They were grown at 30°C and 250 r.p.m. Aliquots were taken at different times for analysis of growth (OD_600_), beta-galactosidase activity, lactose and ethanol.

### Beta-galactosidase activity

Beta-galactosidase activity was measured using the method of Guarente (1983) as previously described (Becerra *et al*., 2001). One enzyme unit (EU) was defined as the quantity of enzyme that catalyses the liberation of 1 nanomol of orthonitrophenol from orthonitrophenyl-β-D-galactopyranoside per min under assay conditions. EU are expressed per ml of culture medium.

### Protein determination

Protein concentration was measured by the method of Bradford (1976) using bovine serum albumin as a standard.

### Other procedures

Standard procedures for manipulation of nucleic acids were essentially those of Sambrook and colleagues (1989). *Escherichia coli* DH-10B was used for plasmid amplification. Yeast transformation was performed using a lithium acetate procedure (Gietz and Woods, 1994).

### Statistical analysis

Data are expressed as mean ± standard deviation. The statistical significance of differences found between means was evaluated by the unpaired Student's *t*-test, two-tailed *P* values calculated with Excel software.
